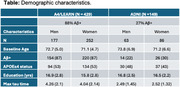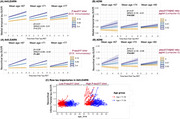# Elevated *p*‐Tau217 is Strongly Associated with Longitudinal Neocortical Tau‐PET in Younger Adults: Findings from the A4/LEARN studies and ADNI

**DOI:** 10.1002/alz70856_106874

**Published:** 2026-01-08

**Authors:** Gillian T Coughlan, Hannah M Klinger, Mabel Seto, Colin Birkenbihl, Annie Li, Michelle E. Farrell, Emma G Thibault, Michael J. Properzi, Aaron P. Schultz, Diana L Townsend, Oliver Langford, Jasmeer P. Chhatwal, Hyun‐Sik Yang, Rema Raman, Michael C. Donohue, Keith A. Johnson, Reisa A. Sperling, Rachel F. Buckley

**Affiliations:** ^1^ Massachusetts General Hospital, Harvard Medical School, Boston, MA, USA; ^2^ Department of Neurology, Massachusetts General Hospital, Harvard Medical School, Boston, MA, USA; ^3^ The Athinoula A. Martinos Center for Biomedical Imaging, Department of Radiology, Massachusetts General Hospital, Boston, MA, USA; ^4^ Alzheimer's Therapeutic Research Institute, University of Southern California, San Diego, CA, USA; ^5^ Massachusetts General Hospital and the Athinoula A Martinos Center for Biomedical Imaging, Boston, MA, USA; ^6^ Brigham and Women's Hospital, Boston, MA, USA; ^7^ Department of Neurology, Harvard Medical School, Boston, MA, USA; ^8^ Alzheimer's Therapeutic Research Institute, Keck School of Medicine, University of Southern California, San Diego, CA, USA; ^9^ Brigham and Women's Hospital, Harvard Medical School, Boston, MA, USA; ^10^ Melbourne School of Psychological Sciences, University of Melbourne, Melbourne, VIC, Australia

## Abstract

**Background:**

The pathophysiological cascade of Alzheimer's Disease(AD) is characterized by amyloid‐β(Aβ) related secretion of soluble phosphorylated tau (*p*‐tau), followed by aggregation of tau tangles and subsequent cognitive decline. Previous literature suggests that tau proliferation is more aggressive at younger ages, but the extent to which this is true and can be detected by plasma markers remains unclear. We examined whether age interacts with plasma *p*‐tau217 and *p*‐tau217/Aβ42 and to predict rates of regional tau‐PET accumulation in baseline clinical normal adults.

**Method:**

Participants were 578 clinically normal individuals (mean age 72.2; 295 *APOE*ε4‐carriers [56%]; mean years of education 16.3) with baseline plasma *p*‐tau217 and longitudinal tau‐PET from the Anti‐Amyloid Treatment in Asymptomatic Alzheimer's Disease (A4) trial and the companion Longitudinal Evaluation of Amyloid Risk and Neurodegeneration (LEARN) study as well as the Alzheimer's Disease Neuroimaging Initiative.

Medial temporal lobe (amygdala, parahippocampal gyrus, entorhinal cortex) and neocortical (inferior parietal, fusiform, inferior temporal) tau composites were chosen as longitudinal tau‐PET outcomes. The average tau‐PET follow‐up time was 3.5 years (SD=1.6 years, range=1.3‐7.1 years). Random‐effects regression estimated the interaction between baseline age and baseline *p*‐tau217 longitudinal tau composites, adjusting for sex and years of education (including participant‐specific intercepts and slopes).

**Result:**

In A4/LEARN, age moderated the association between plasma *p*‐tau217 and neocortical tau accumulation (β=‐0.06, =‐0.09 ‐ ‐0.04, *p* <0.001; Figure 1A), such that the effects of elevated *p*‐tau217 on tau were stronger at younger ages. Similarly in ADNI, age moderated the association between *p*‐tau217/Aβ42 and neocortical tau accumulation (β=‐0.12, =‐0.18 ‐ ‐0.06, *p* <0.001;Figure 1B, such that the effects of elevated *p*‐tau217/Aβ42 on tau accumulation were stronger at younger ages. The raw tau trajectories for one neocortical region as a function of age and *p*‐tau217 is illustrated in Figure 1C. In *Post‐hoc* analysis covarying *APOE*ε4 status, the interactive effects of age and *p*‐tau217 remained significant.

**Conclusion:**

There is evidence for more aggressive tau‐related processes in younger individuals. These findings highlight the critical need for early identification and enrollment in AD clinical trials, potentially using plasma biomarkers in primary care and secondary care.